# Loss of VHL in RCC Reduces Repair and Alters Cellular Response to Benzo[a]pyrene

**DOI:** 10.3389/fonc.2013.00270

**Published:** 2013-10-28

**Authors:** Marten A. Schults, Yvonne Oligschlaeger, Roger W. Godschalk, Frederik-Jan Van Schooten, Roland K. Chiu

**Affiliations:** ^1^Department of Toxicology, Maastricht University Medical Centre, NUTRIM-School for Nutrition, Toxicology and Metabolism, Maastricht University, Maastricht, Netherlands; ^2^Department of Radiation Oncology, University Medical Center Groningen, University of Groningen, Groningen, Netherlands

**Keywords:** nucleotide excision repair, metabolism, carcinogens, renal-cell carcinoma, von Hippel-Lindau

## Abstract

Mutations of the von Hippel-Lindau (*VHL*) tumor suppressor gene occur in the majority of sporadic renal-cell carcinomas (RCC). Loss of *VHL* function is associated with stabilization of hypoxia-inducible factor α (HIFα). We and others demonstrated that there is a two-way interaction between the aryl hydrocarbon receptor, which is an important mediator in the metabolic activation and detoxification of carcinogens, and the HIF1-pathway leading to an increased genetic instability when both pathways are simultaneously activated. The aim of this study was to investigate how environmental carcinogens, such as benzo[a]pyrene (BaP), which can be metabolically activated to BaP-7,8-diOH-9,10-epoxide (BPDE) play a role in the etiology of RCC. We exposed *VHL*-deficient RCC4 cells, in which HIFα is stabilized regardless of oxygen tension, to 0.1 μM BaP for 18 h. The mutagenic BPDE-DNA adduct levels were increased in HIFα stabilized cells. Using qRT-PCR, we demonstrated that absence of *VHL* significantly induced the mRNA levels of AhR downstream target CYP1A1. Furthermore, HPLC analysis indicated that loss of *VHL* increased the concentration of BaP-7,8-dihydroxydiol, the pre-cursor metabolite of BPDE. Interestingly, the capacity to repair BPDE-DNA adducts in the HIFα stabilized RCC4 cells, was markedly reduced. Taken together, these data indicate that loss of *VHL* affects BaP-mediated genotoxic responses in RCC and decreases repair capacity.

## Introduction

Renal-cell carcinoma (RCC) is the most common type of kidney cancer in adults and accounts for 4% of all cancers (1). The most frequently observed genetic alteration in RCC is the somatic mutation of the von Hippel-Lindau (VHL) tumor suppressor gene (2, 3).

The VHL protein (pVHL) is a crucial regulator of the oxygen sensing pathway, which involves the transcription factor hypoxia-inducible factor alpha (HIFα) (2, 4). In the presence of oxygen, HIFα is hydroxylated by an oxygen-dependent prolyl hydroxylase (HIF-PH) (5). An E3 ubiquitin ligase complex containing the pVHL recognizes the hydroxylated HIFα, and targets it for ubiquitination (6) and subsequently proteasomal degradation (7). Under hypoxic conditions, HIFα is not prolyl-hydroxylated and thus unrecognized by pVHL.

The stabilized HIFα can translocate to the nucleus where it forms a heterodimer with HIF1β, also referred to as aryl hydrocarbon receptor nuclear translocator (ARNT) (8). HIF1 then binds to the promoter/enhancer regions in the DNA (9), where it drives the expression of a wide array of hypoxia-inducible genes to augment oxygen delivery or to provide alternative pathways for energy production and cell metabolism.

The functional loss of pVHL in some RCC results in an aberrant stabilization of HIFα independent of the oxygen tension. The subsequent overexpression of proteins encoded by HIFα regulated target genes contributes to the creation of a microenvironment favorable for cell proliferation (2, 10–12).

In many tumors including RCC, the hypoxia-responsive transcription factor HIFα is overexpressed (13), and patients diagnosed with such hypoxic tumors will often have a poor clinical prognosis due to the formation of metastases and the resistance to chemotherapeutics (14). This negative prognosis may occur due to low oxygen concentrations having the capacity to induce genetic instability, leading to increased rates of mutagenesis and angiogenesis, decreased rates of apoptosis and upregulation of genes involved in the metastatic cascade (15). Suppression of the DNA damage response pathways within the hypoxic tumors may also play a critical role (9).

In addition to forming a complex with HIFα, HIF1β/ARNT also dimerizes with the aryl hydrocarbon receptor (AhR) which is known to interact with environmental pollutants such as dioxins and polycyclic aromatic hydrocarbons (PAH). PAHs are widely distributed contaminants produced as byproducts of combustion processes such as in vehicle exhaust, cigarette smoking, and charcoal grilling of food. Benzo[a]pyrene (BaP) is a classic example of PAH and is readily absorbed by inhalation, ingestion, and through the skin. As BaP is lipophilic, it can easily diffuse into cells where it binds to AhR, translocates into the nucleus and subsequently heterodimerizes with HIF1β/ARNT. This complex can then bind to the xenobiotic response elements of target genes (16) where it acts as a transcription factor for a number of genes, which encode for enzymes involved in xenobiotic detoxification, including the cytochrome P450 (CYPs) isoforms *CYP1A1* and *CYP1B1* (17, 18).

The detoxification process of BaP begins with an epoxidation reaction by the mono-oxygenases CYP1A1 and CYP1B1 (phase I). The resulting metabolites (e.g., BaP-7,8-epoxide and BaP-9,10-epoxide) can be converted non-enzymatically to phenols (e.g., 3-OH BaP) or enzymatically to dihydrodiols (e.g., BaP-7,8-diOH or BaP-9,10-diOH) by epoxide hydrolase. Phenols can subsequently be converted to water-soluble sulfate or glucuronide conjugates (phase II) and dihydrodiols can be further transformed by CYP1A1 or CYP1B1 to diol epoxides [e.g., BaP-7,8-diol-9,10-epoxide (BPDE)] or conjugated by uridine diphosphate glucuronosyl transferase (UGT) (18). An unfortunate consequence of the detoxification reaction is the production of the intermediate BPDE, which can covalently bind to DNA forming highly mutagenic DNA adducts (17). When unrepaired, these lesions may result in mutations (19).

Previously, we demonstrated that exposure of cells to hypoxia markedly enhances the genetic instability caused by exogenous genotoxins and that HIF activation decreases nucleotide excision repair (NER) (20). Furthermore, we demonstrated that the kinetics of BaP metabolism is altered under hypoxia resulting in a prolonged time of exposure and a higher amount of BPDE-DNA adducts being formed (Schults et al. manuscript submitted for publication). From these initial studies in which we induced HIFα expression using chemicals or hypoxia, we observed an important link between the HIF1 mediated response pathway and the cellular response pathway that counteracts chemical carcinogens.

In the present study, we hypothesize that in naturally occurring RCC cells that have a defect in HIF regulation, the observed genetic instability may be the result of a faulty response to environmental carcinogens such as BaP. In this current report, we show that loss of *VHL* affects BaP-mediated genotoxic responses by inducing CYP1A1 mRNA levels, which mediated a significant change in BaP-7,8-diOH, the pre-cursor metabolite of BPDE. Secondly, the capacity to repair DNA by NER is reduced in these cells, thereby preventing the repair of those BPDE-DNA adducts.

## Materials and Methods

### Cell culture and treatment

RCC4 (*VHL*^−/−^) cells, a *VHL*-deficient cell line and RCC4-VHL (*VHL*^+/+^), RCC4 reconstituted with *VHL*, were cultured in Dulbecco’s Modified Eagle Medium (DMEM; Sigma-Aldrich, UK) supplemented with 10% heat-inactivated Fecal Calf Serum (FCS; Invitrogen Breda, The Netherlands) and 1% penicillin streptomycin (P/S; Gibco, Invitrogen, Paisley, UK) at 37°C in a 5% CO_2_ and 20% O_2_ atmosphere. Cells were seeded 1 day before treatment and maintained at 37°C in a 5% CO_2_ atmosphere. All cells were treated with 0 or 0.1 μM BaP (Sigma) dissolved in DMSO (final concentrations did not exceed 0.5%) for 18 h. After treatment medium was removed and cells were harvested using trypsin. All samples were stored at −20°C.

### Quantitative real-time PCR

Cells were washed twice with PBS and lysed with Trizol (Invitrogen). Total RNA was isolated according to the manufacturer’s instructions. The quantity of each RNA sample was spectrophotometrically assessed by a Nanodrop 1000 (Thermo Scientific, Waltham, MA, USA). cDNA synthesis was performed using the iScript cDNA Synthesis kit (Biorad, Veenendaal, The Netherlands) starting with 1 μg of RNA. cDNA was diluted 25× in RNase free water. Real-time PCR was performed using the MyiQ Single Color RT-PCR detection system (Biorad) using Sensimix Sybr Green (Quantace, London, UK), 5 μl diluted cDNA and 0.3 μM (Table 1) primers in a total volume of 25 μl. Samples were amplified under the following conditions: 95°C for 3 min, followed by 40 cycles of 95°C for 15 s and 60°C for 45 s. PCR was checked for a-specific products by performing a melting curve analysis (65–95°C). Data were analyzed using the MyiQ Software system (Biorad) and were expressed as relative gene expression (fold increase) using the 2^−ΔΔCt^ method. The stably expressed gene cyclophilin A was included as reference.

**Table 1:** **Primer sequences for real-time RT-PCR**.

Gene		Sequence 5′ → 3′	Accession number
Cyclo- philin a	Forward	TTCCTGCTTTCACAGAATTATTCC	NM_021130.3
	Reverse	GCCACCAGTGCCATTATGG	
CYP1A1	Forward	TCCTGGAGACCTTCCGACACT	NM_000499.3
	Reverse	CTTTCAAACTTGTGTCTCTTGTTGTG	
CYP1B1	Forward	AGTGCAGGCAGAATTGGATCA	NM_000104.3
	Reverse	GCGCATGGCTTCATAAAGGA	
XPA	Forward	CCGACAGGAAAACCGAGAAA	NM_000380.3
	Reverse	TTCCACACGCTGCTTCTTACTG	
XPC	Forward	CCCAGCCCGCTTTACCA	NM_004628.4
	Reverse	TGCATTAACTGTAAATGTTCCAATGA	
ERCC4	Forward	CACCTCCCTCGCCGTGTA	NM_005236.2
	Reverse	CGCAAATATAACACCACCTTGTG	
ERCC5	Forward	GCATGAAATCTTGACTGATATGAAAGA	NM_000123.3
	Reverse	TAAGCAAGCCTTTGAGTTGGTACTG	
ERCC1	Forward	ACCCCTCGACGAGGATGAG	NM_202001.2
	Reverse	CAGTGGGAAGGCTCTGTGTAGA	
GSTP1	Forward	CTCAAAAGGCTTCAGTTGCC	NM_000852.3
	Reverse	ACCTCCGCTGCAAATACATC	
EPHX1	Forward	CTTCACGTGGATGAAGTGGA	NM_000120.3
	Reverse	CTGGCGGAATGAATTTGACT	
UGT1A6	Forward	GGAACCCGACCATCGAATC	NM_001072.3
	Reverse	TCGGGTGACCAAGCAGATC	
UGT2B7	Forward	TCCCATCAAATCTCCACAGA	NM_001074.2
	Reverse	GGTGTTTTCTCTGGGGTCAA	

### DNA isolation

Cells were harvested and resuspended in 400 μl SET/SDS (100 mM NaCl, 20 mM EDTA, 50 mM Tris, 0.5% SDS) and incubated at 37°C for 2 h. About 50 μl of DNAse-free RNAse-solution [RNAse A (0.1 mg/ml) and RNAse T1 (1000 U/ml) in SET, incubated at 80°C for 5 min] was added and samples were incubated at 37°C for 1 h followed by adding 50 μl DNAse-free proteinase K (10 mg/ml in SET-SDS, heat-inactivated at 37°C for 30 min) and samples were incubated overnight at 37°C. After addition of 500 μl phenol/chloroform/Isoamylalcohol (25:24:1), samples were rotated for 5 min and centrifuged for 5 min at 14000 rpm. To the upperphase, 500 μl chloroform/Isoamylalcohol (24:1) was added and samples were 5 min rotated, centrifuged for 5 min (14000 rpm) and 1/30 volume NaAc (3 M, pH 5.2) was added to the upperphase. Samples were mixed for a few seconds and two volumes ethanol 100% (4°C) were added, samples were mixed and incubated at −20°C for 30 min. Samples were centrifuged for 5 min and DNA pellets were washed with ethanol 70%. DNA pellets were dried and resuspended in Mili-Q H_2_O. The quantity and quality of DNA was measured using the Nanodrop 1000.

### ^32^P-postlabeling of BPDE-DNA adducts

DNA adduct levels were determined according to the nuclease P1 enrichment technique originally described by Reddy and Randerath (21) with the modifications described by Godschalk et al. (22). In all experiments, three BPDE-DNA standards with known BPDE-DNA adduct levels (one adduct per 10^6^, 10^7^, and 10^8^ normal nucleotides) were analyzed in parallel for quantification purposes. Adducts spots in DNA from BaP treated RCC4 cells that chromatographed at the same position as the BPDE-DNA adducts standards were considered to be derived from BPDE, and were quantified using Phosphor-Imaging technology (Fujifilm FLA-3000, Rotterdam, The Netherlands).

### HPLC

In order to determine BaP-metabolite composition, 4 ml medium was extracted with 1 ml ethyl acetate after addition of 1 ml 3 M NaCl. Samples were vortexed for 10 min, centrifuged for 5 min at 1000 rpm and after briefly vortexed, centrifuged again for 10 min at 1500 rpm. The ethyl acetate fraction was collected and the extraction step was repeated twice. The second time and third time, only ethyl acetate was added and the third time, also two droplets of ethanol were added to the samples for better separation. The ethyl acetate fractions were combined, evaporated to dryness under N_2_, and residues were resuspended in 0.5 ml methanol. For chromatographic separation, a 50 μl volume of the sample was injected onto a Hypersil 5 μm ODS HPLC column (250 mm × 3 mm i.d.) (Supelco 54933, Bellefonte, PA, USA) at a flow rate of 0.5 ml/min. Mobile phase A and B were 100% methanol (Biosolve Chemicals, Valkenswaard, The Netherlands) and 40% methanol in water, respectively. The time program for the multi-step gradient was: 0–5 min: isocratically 40/60 (A/B, v/v), 5–30 min: gradient from 40/60 (A/B, v/v) to 90/10 (A/B, v/v), 30–35 min: isocratically 90/10 (A/B, v/v), 35–37 min: gradient from 90/10 (A/B, v/v) to 40/60 (A/B, v/v), 37–40 min: 40/60 (A/B, v/v). The total run time was 40 min. For quantitation purposes, a standard mix consisting of 50 ng/ml BaP-9,10-diOH, 50 ng/ml BaP-7,8-diOH, and 50 ng/ml 3-OH BaP (Midwest Research Institute, Kansas City, MO, USA) was used. Samples were analyzed on a Gynkotek P580A HPLC system (Separations Analytical Instruments, Hendrik Ido Ambacht, The Netherlands) with a Spark SP830 autosampler (Spark Holland, Emmen, The Netherlands) and a Perkin Elmer LS-30 programmable fluorescence detector (Perkin Elmer, Foster City, CA, USA). Excitation/emission wavelengths were 257/>350 nm. For quantification, the area of each metabolite peak on the chromatogram was determined.

### Measurement of repair capacity

To assess NER capacity in the liver samples, we applied a well characterized and validated modified comet assay (23). This assay measures the ability of NER-related enzymes that are present in cell extracts, to incise substrate DNA containing BPDE-DNA adducts. The substrate nucleoids were prepared from untreated A549 cells (human epithelial lung carcinoma cells) obtained from the American Tissue Culture Collection (ATCC, Rockville, MD, USA). The cells were cultured in DMEM supplemented with 10% heat-inactivated FCS and 1% penicillin/streptomycin and maintained at 37°C in a 5% CO_2_ atmosphere. A549 cells were embedded in LMP agarose on glass microscope slides and subsequently lyses overnight in cold (4°C) lysis buffer (2.5 M NaCl, 0.1 M EDTA, 0.01 M Tris, 0.25 M NaOH plus 1% Triton X-100 and 10% DMSO). The resulting nucleoids were then either exposed to 1 μM BPDE (NCI Chemical Carcinogen Reference Standard Repository, Midwest Research Institute, Kansas City, MO, USA) in PBS or vehicle control (DMSO, 0.5%) for 30 min at 4°C. To prepare protein/enzyme extracts, RCC4 and RCC4-*VHL* cells were harvested and resuspended in buffer A (45 mM HEPES, 0.4 M KCl, 1 mM EDTA, 0.1 mM dithiothreitol, 10% glycerol, adjusted to pH 7.8 using KOH). Resulting aliquots were snap-frozen, thawed again and 30 μl of 1% Triton X-100 in buffer A per 100 μl of extract was added. Protein concentrations were determined by the BioRAD DC protein assay using bovine serum albumin as a standard. Tissue extracts were diluted to a concentration of 1 mg/ml. Next, four volumes of reaction buffer B (45 mM HEPES, 0.25 mM EDTA, 2% glycerol, 0.3 mg/ml BSA, adjusted to pH 7.8 with KOH) were added and 50 μl of the mixture was added to the gel-embedded nucleoids and incubated for 20 min at 37°C. Alkaline treatment (40 min) and electrophoresis (30 min) were conducted as described in the standard comet assay (24). Comets were visualized using a Zeiss Axioskop fluorescence microscope and quantified as tail moment. Samples were tested in two independent incubations within each single experiment. On every slide, 50 cells were analyzed randomly using the Comet assay III software program (Perspective Instruments, Haverhill, UK). The increased DNA strand breakage (tail moment) in the BPDE-modified nucleoids vs. the DMSO-treated nucleoids is indicative for the NER capacity of the cell extracts. The final repair capacity was calculated according to Langie et al. (23).

### Statistical analysis

Results are expressed as the mean ± SE of the mean. GraphPad Prism 4 was used for statistical analysis. A two-way analysis of variance (ANOVA) with Bonferroni *post hoc* multiple comparison correction was used to assess differences in mRNA levels. To analyze differences in, metabolite levels, adduct levels, and repair capacity a Student’s *t*-test was used. Differences were considered to be statistically significant when *P* < 0.05.

## Results

### BPDE-DNA adduct levels are higher in cells deficient in *VHL*

**Figure 1:**
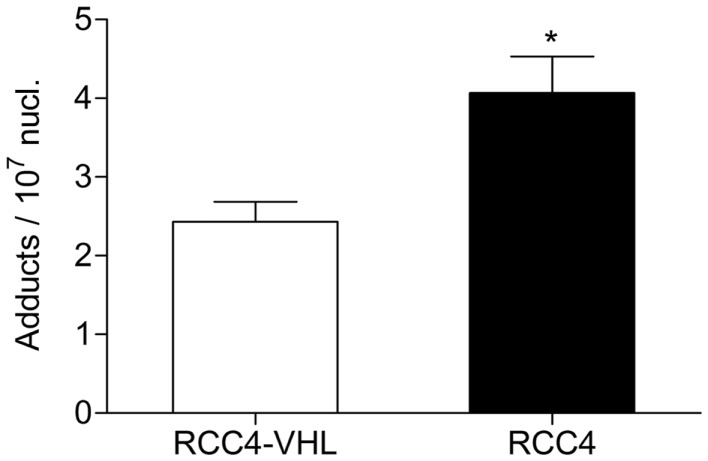
**BPDE-DNA adduct levels are higher in cell deficient in *VHL***. RCC4-*VHL* and RCC4 cells were incubated with 0.1 μM BaP. DNA was isolated after 18 h and BPDE-DNA adduct levels were measured by ^32^P-postlabeling. Data (*n* = 4) are presented as mean adduct level per 10^7^ nucleotides ± SE (*P < 0.05, Student’s *t*-test).

To examine the influence of *VHL* deletion on BaP-mediated genotoxic responses, we quantified the amount of BPDE-DNA adducts in *VHL*-deficient RCC4 cells and compared it with the genetically wild type RCC4-VHL cells and normalized it to the total amount of DNA (Figure 1). Exposure to 0.1 μM BaP resulted in a ∼1.7-fold greater formation of the pro-mutagenic BPDE-DNA adduct in RCC4 cells, compared to RCC4-VHL cells (*P* < 0.05).

### Cells deficient in *VHL* have changes gene expression of metabolic enzymes

**Figure 2:**
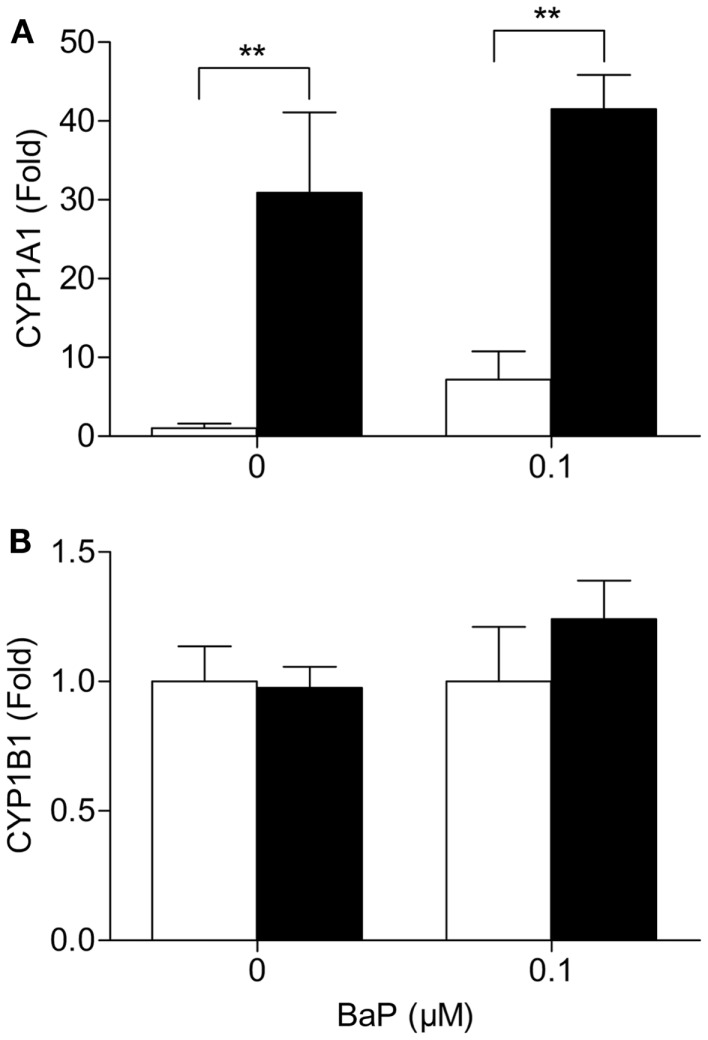
**CYP1A1 but not CYP1B1 mRNA expression, is markedly enhanced in *VHL* defective cells**. RCC4-*VHL* (□) and RCC4 cells (■) were incubated with DMSO or 0.1 μM BaP for 18 h. RNA was isolated and mRNA levels were measured of **(A)** CYP1A1 and **(B)** CYP1B1. Data (*n* = 4) are presented as mean fold change ± SE (**P < 0.01).

**Figure 3:**
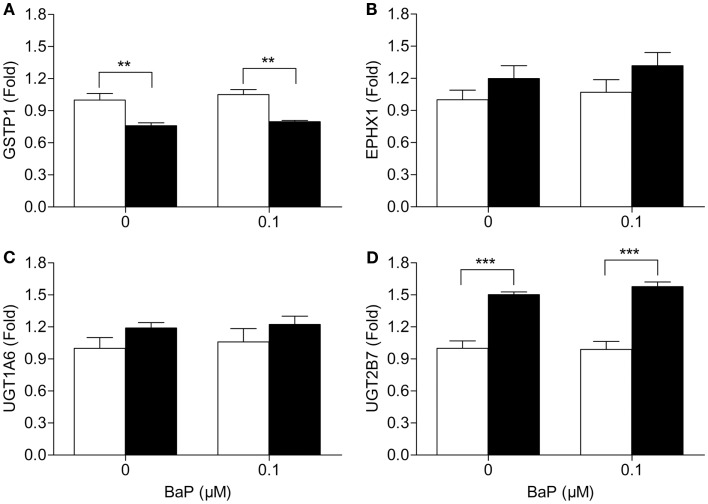
**Loss of *VHL* increases UGT2B7, but decreases GSTP1 gene expression**. RCC4-*VHL* (□) and RCC4 cells (■) were incubated with DMSO or 0.1 μM BaP for 18 h. RNA was isolated and mRNA levels were measured of **(A)** GSTP1, **(B)** EPHX1, **(C)** UGT1A6, and **(D)** UGT2B7. Data (*n* = 4) are presented as mean fold change ± SE (**P < 0.01, ***P < 0.001, two-way ANOVA with Bonferroni *post hoc* multiple comparison correction).

Since metabolism may be the underlying cause of the increase in BPDE-DNA adducts in cells deficient in *VHL*, we assessed the mRNA expression of several key phase I and II metabolic enzymes. Of the phase I enzymes responsible for the activation of BaP, CYP1A1 mRNA levels significantly differ between the two cell lines (Figure 2A). The CYP1A1 mRNA levels were ∼31 and ∼5.8 times higher in the RCC4 cells compared to the RCC4-*VHL* cells (*P* < 0.01), for untreated and BaP treated cells, respectively. CYP1B1 gene expression was not statistically different between the two cell lines (Figure 2B). Gene expression of four phase II enzymes responsible for the conjugation of BaP metabolites was also measured. The expression of the glutathione S-transferases GSTP1 was ∼24% lower in RCC4 cells compared to RCC4-VHL cells (*P* < 0.05, Figure 3A). Epoxide hydrolase 1 (Figure 3B) and the UDP glucuronosyltransferases UGT1A6 (Figure 3C) showed no difference between the two cell lines. Conversely, UGT2B7 expression was ∼1.5 and 1.6 times higher (DMSO and BaP treatment respectively) in RCC4 cells compared to the RCC4-VHL cells (Figure 3D).

### Absence of *VHL* directs BaP metabolism toward unfavorable activation

**Figure 4:**
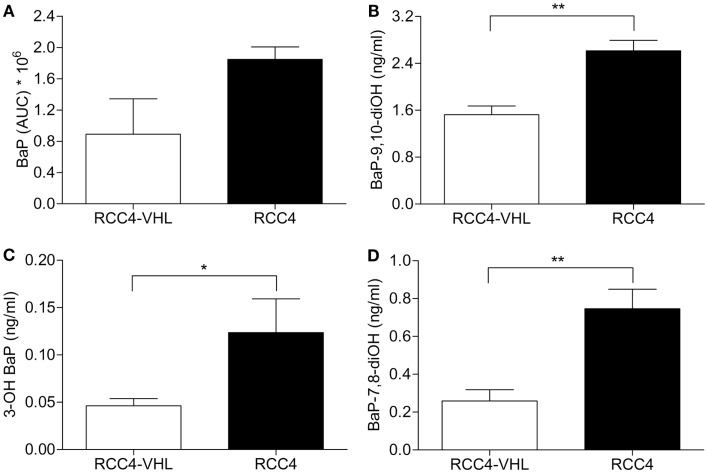
**Absence of *VHL* decreased BaP metabolism, but increased BaP metabolites**. RCC4-*VHL* (□) and RCC4 cells (■) were incubated with 0.1 μM BaP for 18 h and extracellular unmetabolized BaP **(A)**, BaP-9,10-diOH **(B)**, BaP-7,8-diOH **(C)**, and 3-OH BaP **(D)** metabolites were measured. Data (*n* = 5) are presented as mean area under the curve ± SE (*P < 0.05, **P < 0.01; Student’s *t*-test).

To determine whether the observed differences in BaP metabolic enzymes between the two cell lines resulted in the expected detrimental changes in BaP metabolism, we assessed BaP and its metabolites BaP-9,10-diOH, BaP-7.8-diOH and 3-OH BaP by HPLC analysis with fluorescence detection. RCC4 and RCC4-VHL were exposed for 18 h to 0.1 μM BaP and the medium was collected. The rate of metabolism was determined from measuring the remaining amount of unmetabolized BaP. In the medium, a non-statistically significant ∼twofold higher level of BaP was measured in the RCC4 cells compared to the control RCC4-VHL cells (Figure 4A). For all three metabolites assessed, statistically significant higher levels of metabolites were found in the medium of the RCC4 cells compared to the RCC4-VHL cells. A ∼1.7- and 2.7-fold induction of BaP-9,10-diOH and 3-OH BaP, respectively, was observed in RCC4 cells compared to RCC4-VHL cells (Figures 4B,C). Furthermore, compared to RCC4-VHL cells, BaP-7,8-diOH levels, the BPDE pre-cursor, were ∼2.9 times higher in RCC4 cells (Figure 4D).

### Nucleotide excision repair capacity is reduced in *VHL*-deficient cells

**Figure 5:**
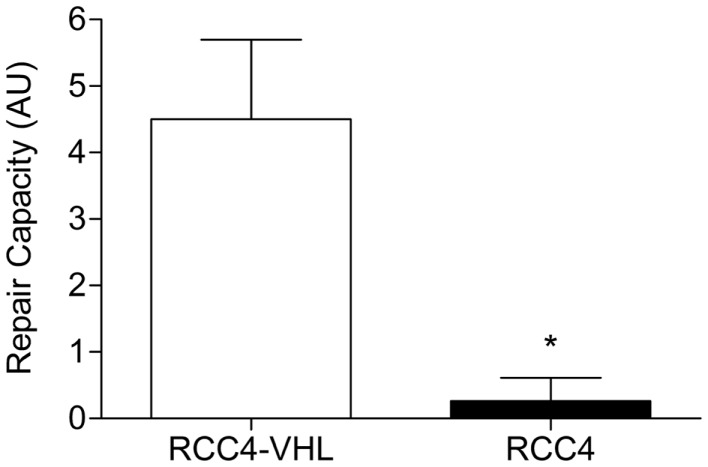
**NER capacity is decreased in RCC4 cells**. The ability to repair BPDE-DNA adducts in RCC4-*VHL* and RCC4 cells were measured using the modified comet assay (23). Data (*n* = 4) are presented as mean fold change ± SE (*P < 0.05, Student’s*t*-test).

As we previously reported a downregulation of NER capacity in HIFα stabilized cells, we sought to determine whether diminished DNA repair may also play a role in the observed differences in BPDE-DNA adduct levels induction between the RCC4 and RCC4-VHL cells. Firstly, we determined the influence of DNA repair gene expression in the matched cells. The mRNA levels of the critical NER genes *XPA, XPC, ERCC1, ERCC5*, and *ERCC4* were not altered in the cell lines (Table 2). Secondly, as DNA repair is often not regulated at the transcription level, we used a validated modified comet assay to determine the functional NER capacity. A markedly reduced repair capacity was observed in the *VHL*-deficient cells compared to the reconstituted RCC4-VHL cells (Figure 5).

**Table 2:** **Relative NER gene expression**.

BaP		XPA	XPC	ERCC4	ERCC5	ERCC1
0 μM	RCC4-*VHL*	1.00 ± 0.02	1.00 ± 0.14	1.00 ± 0.08	1.00 ± 0.10	1.00 ± 0.09
	RCC4	0.94 ± 0.05	0.98 ± 0.12	0.98 ± 0.03	1.16 ± 0.11	1.09 ± 0.06
0.1 μM	RCC4-*VHL*	1.02 ± 0.04	1.04 ± 0.16	0.95 ± 0.08	0.98 ± 0.08	1.01 ± 0.02
	RCC4	1.15 ± 0.05	1.17 ± 0.19	0.89 ± 0.02	1.21 ± 0.12	1.17 ± 0.09

*Statistical method used: two-way ANOVA with Bonferroni *post hoc* multiple comparison correction*.

## Discussion

Previously, we demonstrated that the stabilization of HIFα by CoCl_2_ enhanced the carcinogenic effect of BaP in lung cancer cells and reduced repair (20). Furthermore, we demonstrated that the kinetics of carcinogen metabolism altered under hypoxic conditions, resulting in more BPDE-DNA adducts being formed (Schults et al. manuscript submitted for publication). The aim of the current study was to determine whether similar genetic instability mechanisms hold true in the naturally occurring *VHL*-deficient RCC cells. In this report, we demonstrate that the loss of *VHL* and via presumably the stabilization of HIFα, affects both genetic stability related processes of BaP-mediated and DNA repair capacity in RCC cells.

Cytochrome P450 enzymatically converts BaP into BPDE. This active metabolite subsequently binds DNA covalently forming highly mutagenic DNA adducts (17). To investigate the effect of metabolically activated BaP on RCC4 cells, the formation of BPDE-DNA adducts was determined. Our data demonstrated that *VHL* deficiency resulted in increased BPDE-DNA adduct levels in RCC4 cells, compared to RCC4-VHL cells, when treated with 0.1 μM BaP. This is in agreement with our previously observed results, where the formation of BPDE-DNA adducts was increased due to stabilization of HIFα by CoCl_2_ in A549 cells (20).

To determine whether metabolism may be the cause of the increased BPDE-DNA adduct levels in VHL-deficient cells changes in gene expression of relevant enzymes in the metabolism of BaP were assessed. CYP1A1 and CYP1B1 are two of the most important enzymes in the detoxification and bioactivation of BaP (17, 18). Furthermore, CYP1A1 is the critical enzyme in the bioactivation of BaP-7,8-diOH to the ultimate carcinogenic BPDE metabolites in human lung cell lines *in vitro* (25). In this study, we demonstrate that CYP1A1 mRNA levels were strongly upregulated in the HIFα stabilized RCC4 cells compared to the reconstituted genetically wild type cells. As CYP1B1 mRNA levels were unaffected by *VHL* loss, CYP1A1 appears to be the critical player in RCC metabolism of BaP in these cells. Induction of CYP1A1 in HIFα-stabilized cells may result in an increased level of BPDE formation, which may subsequently induce carcinogenicity. This observation compares well with studies in other cell types where CYP1A1 activity has been positively correlated with pulmonary PAH (BPDE)-associated DNA adduction (26), and high CYP1A1 inducibility in lymphocytes has been related to a high lung cancer risk (27, 28).

The resulting metabolites of BaP can be converted into hydrophilic products by phase II enzymes (18). Alterations in these conjugation reactions result in changed elimination of these compounds and subsequently BPDE-DNA adducts level. No pattern was observed for phase II enzymes, suggesting that they are not likely involved in the observed different BPDE-DNA adduct levels identified between the two cell lines. Nevertheless, measuring BaP metabolites provides a better insight into differences in BaP metabolism between the two RCC4 cell lines. BaP-9,10-diOH was the most abundant metabolite present in the media of both cell lines. This metabolite subsequently forms BaP-9,10-diol-7,8-epoxide, which importantly is far less carcinogenic than BPDE. We further observed that the increased levels of metabolites and in particular BaP-7,8-diOH, which can be oxidized to BPDE, correlates well with the observed increased DNA adducts levels. Together, these data indicate that *VHL*-deficient RCC cells are able to differentially bioactivate BaP as compared to the reconstituted RCC4-VHL cells suggesting a plausible explanation for the increased DNA adduct formation in RCC4 cells.

The level of DNA adducts is influenced by the balance between the induction of the damage and its repair. Therefore, in addition to the formation of adducts, we further analyzed whether loss of *VHL* influences NER since the bulk of adducts are removed by this DNA repair pathway (29). Our data indicate that the loss of *VHL* had no effect on DNA repair gene expression. As DNA repair is often regulated in multiple levels in addition to transcriptional control, we sought to determine whether NER is functionally impaired by HIFα stabilization. To determine the functionality of NER, we used a previously validated modified comet assay, which predominantly assesses the cellular capacity in the recognition and incision phase of NER to remove bulky DNA adducts (23). A dramatically lower repair capacity was observed in the *VHL*-deficient RCC4 cells. Reduced NER capacity under hypoxic conditions was previously demonstrated in mouse fibroblasts (30) and in HIFα stabilized A549 cells (20). In the present study, we show that lack of *VHL* in cells also results in a decreased repair capacity. The reduced NER repair further explains the observed higher BPDE-DNA adduct levels in *VHL*-deficient RCC cells.

In this study, we provide evidence that loss of *VHL* presumably via the stabilization of HIF affects the BPDE-DNA adduct levels in RCC cells and is in fact a double edged sword. Firstly, the absence of *VHL* is associated with induced CYP1A1 mRNA levels, which mediated a significant change in BaP-7,8-diOH levels among other metabolites. This could significantly induce the formation of BPDE-DNA adduct levels. Secondly, the capacity to repair DNA by NER is reduced in HIFα stabilized cells, thereby preventing the repair of those BPDE-DNA adducts. Taken together, these data indicate that loss of *VHL* increases carcinogen genotoxicity in RCC *in vitro* and provides potential insight in the malignant progression into RCC.

## Conflict of Interest Statement

The authors declare that the research was conducted in the absence of any commercial or financial relationships that could be construed as a potential conflict of interest.
